# Identification of an exporter that regulates vitamin C supply from blood to the brain

**DOI:** 10.1016/j.isci.2021.103642

**Published:** 2022-01-13

**Authors:** Hiroshi Miyata, Yu Toyoda, Tappei Takada, Toshimitsu Hiragi, Yu Kubota, Ryuichiro Shigesawa, Ryuta Koyama, Yuji Ikegaya, Hiroshi Suzuki

**Affiliations:** 1 Department of Pharmacy, The University of Tokyo Hospital, 7-3-1 Hongo, Bunkyo-ku, Tokyo 113-8655, Japan; 2 Laboratory of Chemical Pharmacology, Graduate School of Pharmaceutical Sciences, The University of Tokyo, 7-3-1 Hongo, Bunkyo-ku, Tokyo 113-0033, Japan

**Keywords:** Biological sciences, Biochemistry, Molecular biology

## Abstract

Vitamin C (VC) distribution in our body requires VC transporters. However, mammalian VC exporters are yet to be identified. Herein, to unravel this long-standing mystery, we focused on the pathways whereby VC moves from blood to the brain, which should require a VC entrance and exit system composed of an importer and a latent exporter. Via cell-based transport analyses of VC efflux and using knockout mice generated via the CRISPR-Cas9 system, we identified GLUT12/SLC2A12 as a physiologically important VC efflux protein expressed in the choroid plexus; *Glut12/Slc2a12* knockout halved the cerebral VC levels, markedly increased VC accumulation in the choroid plexus, and reduced the cerebrospinal fluid VC levels. These findings facilitate our understanding of VC regulation and the physiological impact of VC in our body.

## Highlights

• A long-standing mystery in vitamin C handling in mammalians was uncovered
• GLUT12 was identified as a physiologically important vitamin C efflux protein—VCEP
• GLUT12 is expressed in the choroid plexus and acts as a vitamin C exporter
• *Glut12* knockout halved the cerebral vitamin C levels in mice

## Graphical abstract

**Figure:**
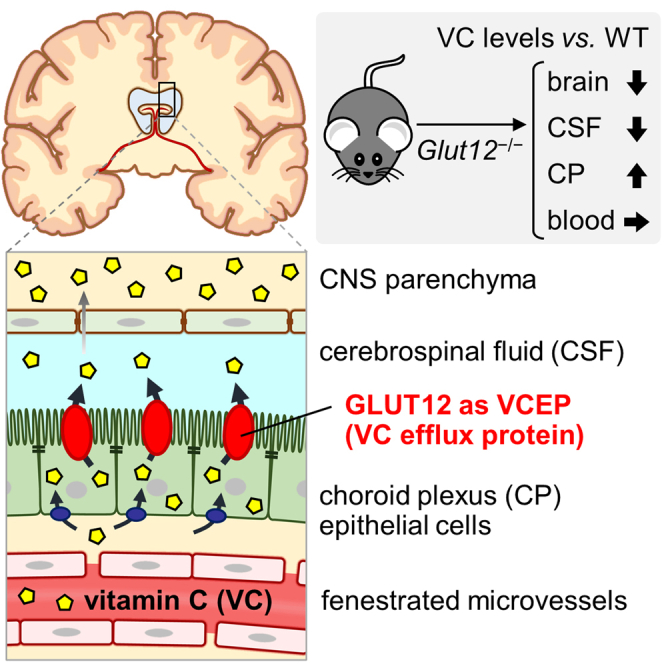

## Introduction

Vitamin C (VC), also known as L-ascorbic acid, is a physiologically important bioactive compound; in humans, VC deficiency leads to scurvy, a disease known to affect ancient sailors on long voyages with minimal VC intake. Humans cannot synthesize VC; therefore, dietary supplementation and subsequent distribution of this water-soluble nutrient are important for the maintenance of human health (Arrigoni and De Tullio, 2002; Figueroa-Mendez and Rivas-Arancibia, 2015). As VC can hardly penetrate cellular membranes passively owing to its hydrophilicity, VC transporters play a pivotal role in VC handling *in vivo*. Until now, only two VC transporters, sodium-dependent vitamin C transporter 1 (SVCT1/SLC23A1) and SVCT2/SLC23A2, have been identified in mammals (Corpe et al., 2010; Sotiriou et al., 2002; Tsukaguchi et al., 1999); they work as sodium-dependent transporters involved in VC uptake from the extracellular space into the cytosol. However, how intracellular VC exits the cells has not been evidenced (Harrison and May, 2009); carrier-mediated VC efflux has only been speculative in nature. Indeed, to the best of our knowledge, human VC exporters that contribute to the cellular secretion of VC on the opposite side of SVCTs for transcellular VC transport have not yet been reported.

Herein, to identify latent VC exporter(s), we have focused on a delivery route of VC from blood to the brain, a VC-abundant tissue in the body, in the blood-brain barrier and the blood-cerebrospinal fluid (CSF) barrier. Historically, the penetration of VC from blood into the CSF through the choroid plexus (CP), a physiologically important process in VC handling (Harrison and May, 2009; Rice, 2000; Spector and Lorenzo, 1973), was identified in 1966 via whole-body radioautography investigations (HammarstrÖM, 1966); however, the set of molecular entities controlling this route has been a long-standing mystery, except for SVCT2, which reportedly transports VC from blood into the cytosol on the basal (blood) side of the plasma membrane in CP epithelial cells (the main component of the blood-CSF barrier) (Sotiriou et al., 2002; Ulloa et al., 2019). In fact, how the incorporated VC passes through the apical (CSF) side of the CP epithelial cells remains unknown, which led us to identify the VC efflux protein (VCEP) that should be involved in the regulation of brain VC levels potentially linking with the risk of cerebral disorders.

## Results and discussion

### GLUT12 is a VC transporter

First, we focused on the members of the glucose transporter (GLUT)/SLC2A family of transporters. Considering that GLUT1/SLC2A1 and GLUT3/SLC2A3 were identified as dehydroascorbic acid (DHA, an oxidized form of VC) transporters (Banhegyi et al., 2014; Rumsey et al., 1997), and that GLUT family proteins can not only transport glucose but also other sugars and urate (an anionic form of uric acid) (Augustin, 2010), we hypothesized that some GLUT proteins function as VC transporters. In addition, GLUT proteins generally act as bidirectional transporters, suggesting that they function as latent VC exporters.

Hence, using *in situ* hybridization image data obtained from the Allen Mouse Brain Atlas (https://mouse.brain-map.org/) (Lein et al., 2007), we investigated the expression of *Glut* genes in the CP of the murine brain. As shown in Figure S1A, *Svct2* was expressed in the CP of the inferior horn of the lateral ventricle and the fourth ventricle, similar to *transthyretin* (*Ttr*), a CP marker. Among 11 *Gluts*, data for which are available (Table S1), only *Glut12* was expressed in the CP. Therefore, we assessed the cerebral expression of *Glut12* (Figure S1B). As expected, both *Glut12* and *Svct2* were expressed in the CP, which was also supported by the results of a previous study (Dahlin et al., 2009). Similar to that of *Ttr*, *Glut12* mRNA levels in the CP were substantially higher than those in other parts of the brain. Furthermore, when expressed in polarized MDCKII cells, Svct2 was mainly localized on the basolateral membrane, whereas Glut12 was expressed on the apical membrane (Figure S1C), which was consistent with our working hypothesis that Glut12 can act as a cellular exporter of VC, along with Svct2-mediated cellular uptake.

**Figure 1:**
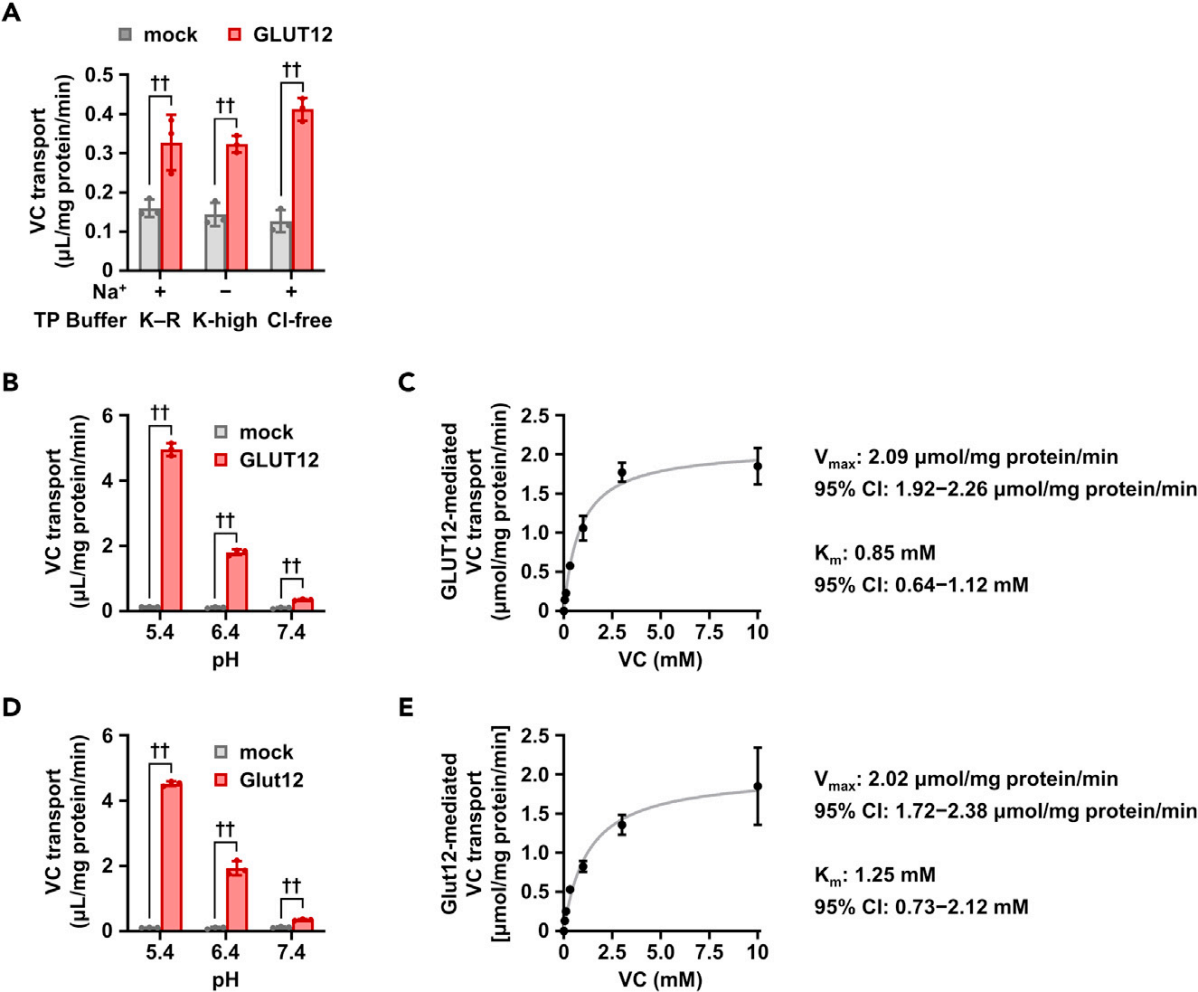
GLUT12 is a VC transporter (A) [1-^14^C]-VC transport activities of GLUT12 transiently expressed in HEK293 cells in various transport buffers in pH 7.4. (B–E) Acidic pH- (B and D) and concentration- (C and E) dependent [1-^14^C]-VC transport activities of human GLUT12 and mouse Glut12 in Krebs-Ringer (K–R) buffer, pH 5.4 (unless otherwise indicated). Data show mean ± SD, *n* = 3. ^††^*P* < 0.01 (two-sided *t* test).

To determine whether human GLUT12 and mouse Glut12 can transport VC, we conducted a cell-based VC uptake assay using HEK293 cells (a versatile cell line with high transfection efficiency and low background for VC uptake) transiently expressing GLUT12 or Glut12 (Figure 1). Prior to the assay, we first confirmed that GLUT12 and Glut12 were functionally expressed in the assay system (Figures S2A and S2B). Subsequently, the cellular activities for GLUT12- or Glut12-mediated [1-^14^C]-VC incorporation were studied as described below.

With GLUT12, VC transport was detected both in Krebs-Ringer buffer mimicking the ionic content of plasma and a sodium-free high-potassium buffer condition that depolarizes cellular plasma membranes (Figure 1A); this indicated that GLUT12 is a sodium-independent and bi-directional VC transporter. Furthermore, imposition of a chloride gradient via the complete removal of external chloride negligibly affected VC transport activity. In addition, the VC-transporting activity of GLUT12 was high at low pHs (Figure 1B). Subsequently, to determine the kinetic parameters, we investigated concentration-dependent VC uptake at 5 min, which enabled us to determine the initial rate of VC uptake by GLUT12 in a time-course experiment, consistent with our previous study on GLUT12-mediated urate transport (Toyoda et al., 2020a). The GLUT12-mediated VC transport could be saturated (Figure 1C); the estimated Michaelis constant (*K*_m_) and maximum velocity (*V*_max_) for VC were 0.846 mM and 2.085 μmol/min per mg protein, respectively. However, given physiologically relevant VC concentrations in human CSF (several hundred micromoles) (Travica et al., 2020) and the steady-state plateau VC concentrations in human plasma (approximately 50–90 μM in healthy subjects with ≥100 mg of daily VC intake) (Levine et al., 1996), together with the fact that these concentrations are considerably lower than the determined *K*_m_, GLUT12 appears to keep its VC transport property independent of VC levels in the CSF and blood.

Similar results were obtained with mouse Glut12 (Figures 1D and 1E). The Glut12-mediated VC transport was also saturated; the estimated *K*_m_ was 1.245 mM, suggesting that the affinities of mouse and human GLUT12 for VC were comparable (Figure 1E). Considering that the reported *K*_m_ values of VC transport via SVCT2 ranged from 10 to 100 μM irrespective of the species (humans or rodents) (Savini et al., 2008), GLUT12 appears to possess a higher capacity for VC transport than SVCT2. This balance may be reasonable considering the hypothesized GLUT12-mediated VC export from cells harboring higher VC levels, which are generated by SVCT2 in an energy-dependent manner against the concentration gradient, compared with that in blood. In other words, given the observed apical localization of Glut12 in polarized cells (Figure S1C), GLUT12 appears to be involved in the smooth secretion of VC into the CSF and preferential supply of VC to the brain, coupled with constant VC uptake from the blood via the SVCT2 entrance machinery.

### Glut12 can act as a VC exporter

**Figure 2:**
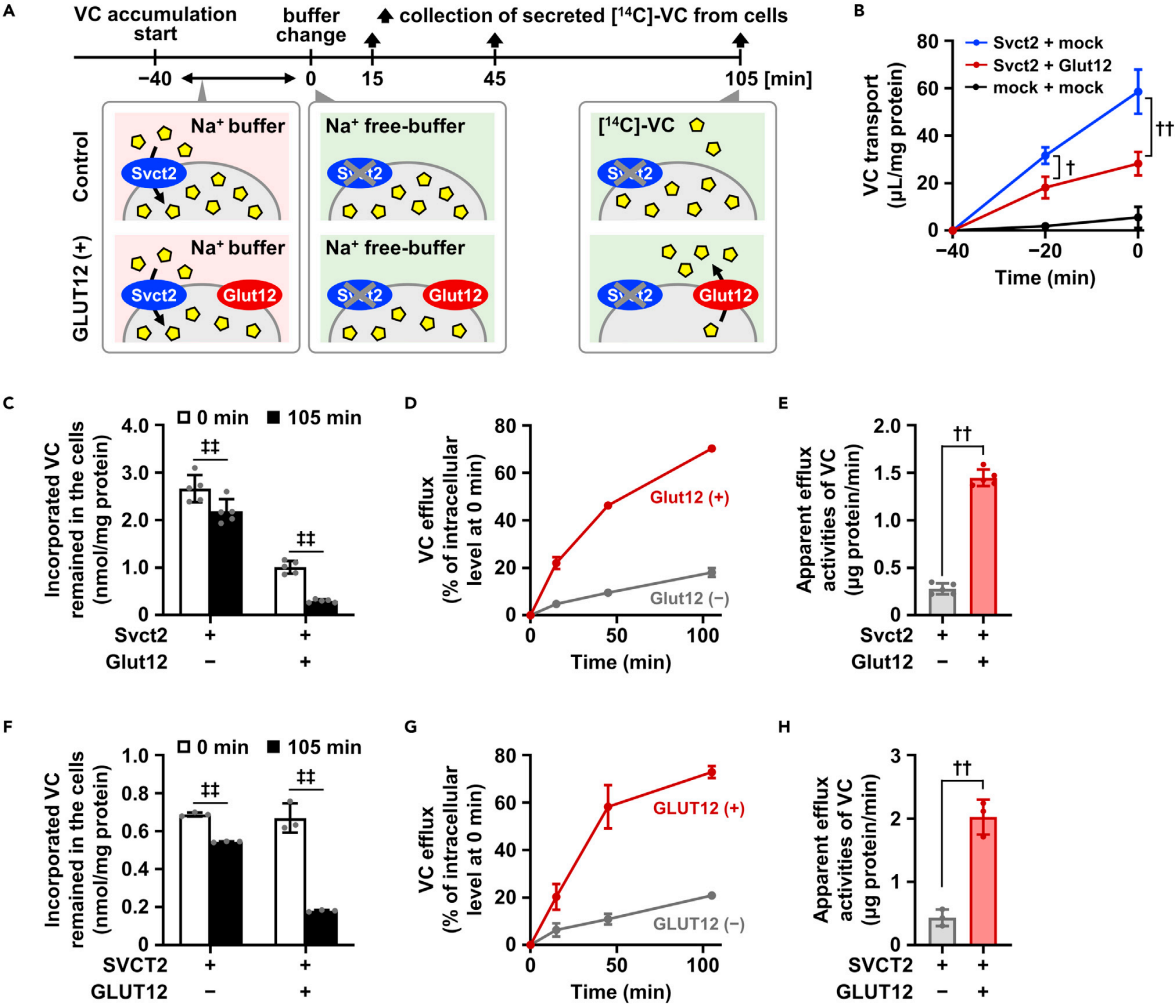
Glut12 can act as a VC exporter (A) Schematic illustration of [1-^14^C]-VC efflux assay. VC accumulation and efflux were conducted in each buffer condition at pH 7.4. (B–E) Forty-eight hours after plasmid transfection, HEK293 cells transiently expressing mouse Glut12 and mouse Svct2, which was employed for the cellular accumulation of [1-^14^C]-vitamin C (VC), were used for a VC efflux assay. (B) Svct2-mediated [1-^14^C]-VC accumulation prior to the efflux assay. (C) [1-^14^C]-VC levels remaining in the cells at the start and end of the efflux phase (0–105 min). A two-factor factorial ANOVA showed the significant effect of Glut12 without significant two-factor interaction. (D) Time-dependent [1-^14^C]-VC efflux from cells with or without Glut12 expression. (E) Cellular [1-^14^C]-VC efflux activities in the absence or presence of Glut12. (F–H) HEK293 cells transiently expressing human GLUT12 and human SVCT2 were used for a VC efflux assay. (F) [1-^14^C]-VC levels remaining in the cells. (G) Time-dependent [1-^14^C]-VC efflux from cells with or without GLUT12 expression. (H) Cellular [1-^14^C]-VC efflux activities in the absence or presence of GLUT12. Data show mean ± SD, *n* = 3 (B), 5 (C–E), 3 (F–H). ^†^*P* < 0.05, ^††^*P* < 0.01 (two-sided *t* test); ^‡‡^*P* < 0.01 (paired *t* test). See also Figures S2.

Next, prior to *in vivo* experiments addressing the latent physiological impact of directional VC transport mediated by Glut12, we investigated whether Glut12 exports VC from the cells. To test this, we constructed a cell-based VC efflux assay system in which cellular accumulation of [1-^14^C]-VC was achieved in a Svct2-dependent manner, followed by the measurement of radioactivity secreted from the cells into a fresh VC- and sodium-free culture medium, a condition that does not allow Svct2 to function as the VC uptake machinery (Figure 2A). In the uptake stage, co-transduction of Glut12 decreased the apparent activity of VC incorporation mediated by Svct2 (Figure 2B). Considering the bidirectionality in the transporter function of GLUTs, this result implied that Glut12-mediated VC efflux coordinated with Svct2-mediated cellular accumulation of VC.

In the secretion stage, Glut12 markedly accelerated VC efflux from the cells, as expected (Figures 2C–2E), indicating that Glut12 can act as a VC exporter. Indeed, in the Glut12-expressing cells, remaining VC decreased more drastically (Figure 2C) and time-dependent VC efflux was faster (Figure 2D) than in control cells; apparent VC efflux activities, calculated as a trend over 0–45 min in Figure 2D, were increased by 5.1-fold in the presence of Glut12 (Figure 2E). After the confirmation of the cellular function of SVCT2 (Figure S2C), we also successfully identified the VC efflux activity of GLUT12 using a similar system expressing both GLUT12 and SVCT2 (Figures 2F–2H).

### Glut12 plays a pivotal role in active transport process of VC from blood to the CSF

**Figure 3:**
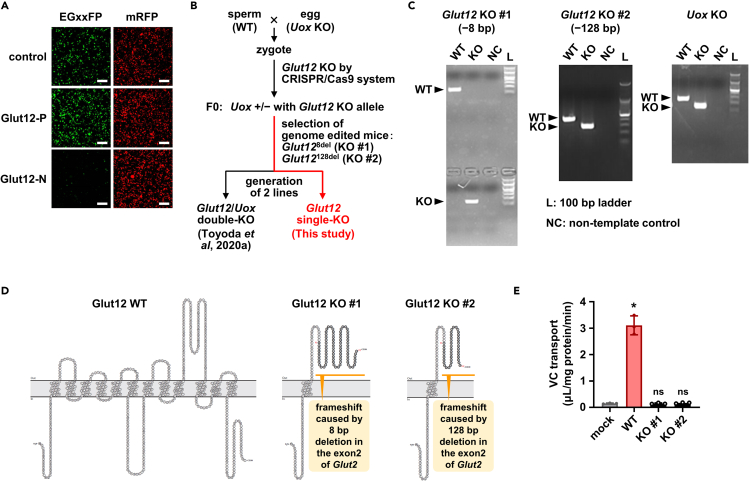
Generation and validation of *Glut12* KO mice (A) *In vitro* evaluation of sgRNAs for *Glut12* KO with an EGxxFP system. Control, previously validated sgRNA sequence targeting Centrin 1 as a positive control for the EGxxFP system; Glut12-P, sgRNA sequence targeting Glut12 worked best in this study (5′-cctcatcggggcattcctcgcct-3′); Glut12-N, representative sgRNA sequence targeting Glut12 worked poorly in this study (5′-cccagcatgtttacgttcctgac-3′). Scale bars, 200 μm. (B) Schematic illustration of the generation flow of *Glut12* KO mice. (C) Representative results of PCR-based genotyping for each KO allele in *Glut12* KO mice. The far-right lane in each image represents a 100-bp ladder marker. *Uox*, *urate oxidase*; WT, wild-type; KO, knockout; NC, non-template control. (D) Topology models of Glut12 KO mutants with a frameshift induced by genome editing. (E) Functional validation of Glut12 KO mutants. Each Glut12 KO mutant transiently expressed in HEK293 cells was functionally null as a vitamin C transporter in Krebs-Ringer buffer (pH 5.4) including 20 μM [1-^14^C]-VC. Data are mean ± SD, *n* = 3. Statistical analyses for significant differences in each group were performed using Bartlett's test, followed by Dunnett's test (^∗^*P* < 0.05 versus control. ns, not significantly different).

**Figure 4:**
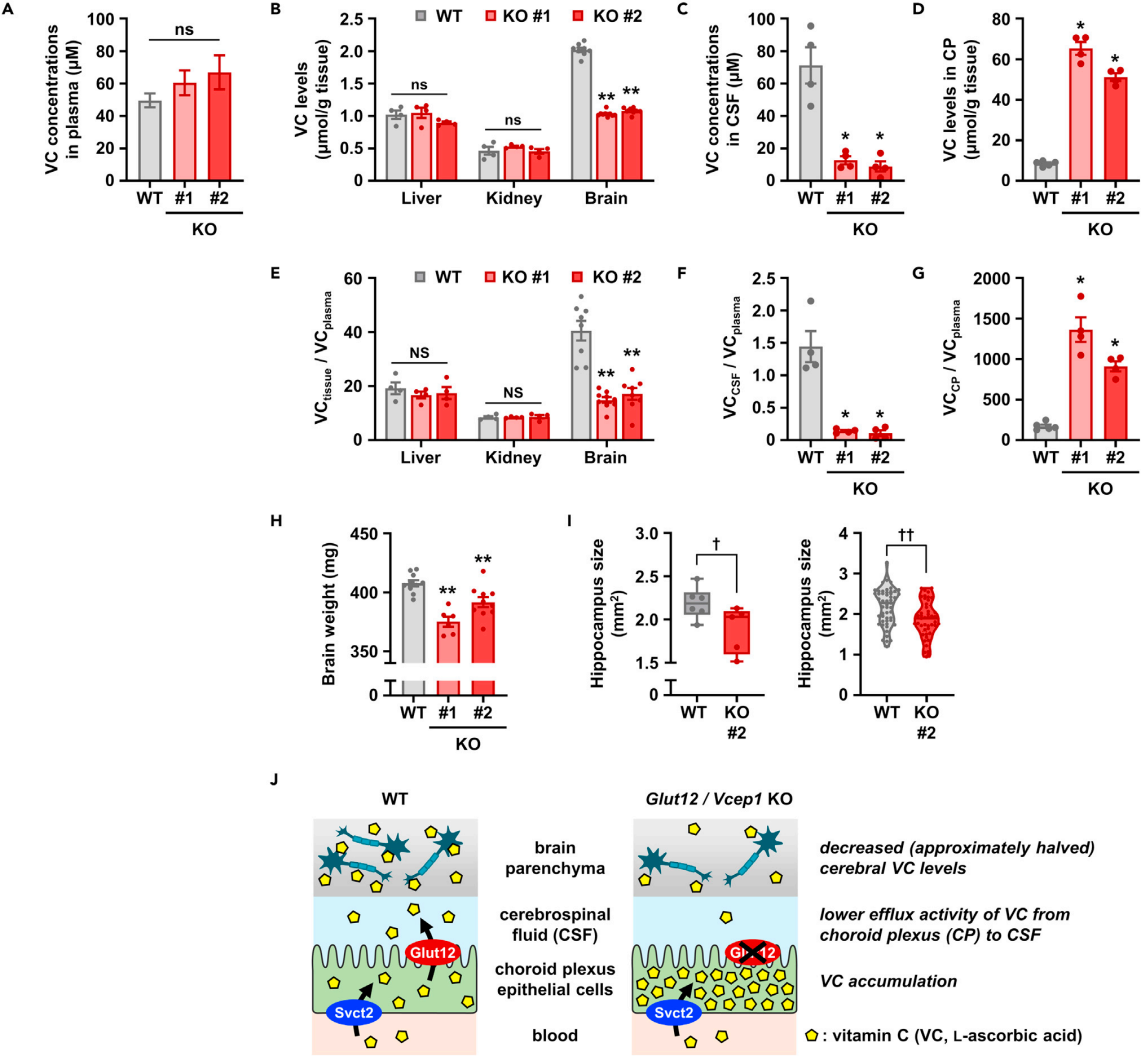
Glut12 plays a pivotal role in active transport process of VC from blood to the CSF (A–D) VC levels in plasma (A), main tissues (B), CSF (C), and CP (D) of *Glut12* KO mice. (E–G) Calculated tissue-to- (E), CSF-to- (F), and CP-to- (G) plasma VC concentration ratios. (H) Reduction in brain weight in *Glut12* KO mice (12–16 weeks of age). Data show mean ± SEM, *n* = 13–15 (A), 4–8 (B–G), and 6–10 (H). ^∗^*P* < 0.05, ^∗∗^*P* < 0.01 versus WT; ns, not significantly different (a parametric Dunnett's test or a non-parametric Steel test). (I) Comparison of hippocampus size between WT and *Glut12* KO #2 mice. *Left*, hippocampus size (dorsal) of each mouse was calculated as the average of calculated areas in several (5–10 per mouse) brain sections, then box-and-whisker plots (minimum to max) were described. *n* = 6 (WT) and 5 (KO #2). *Right*, violin plots were described using all brain sections for hippocampus size analysis. *n* = 51 sections (WT) and 45 sections (KO #2). ^†^*P* < 0.05; ^††^*P*< 0.01 (one-sided *t* test). (J) Proposed model of Glut12 function in the CP as vitamin C efflux protein 1 (Vcep1). See also Figures S3 and S4.

Finally, we investigated the physiological importance of Glut12 with respect to VC handling using two lines (#1 and #2) of *Glut12* knockout (KO) mice generated using the CRISPR-Cas9 system (Figure 3). In this study, all mice studied were males. Endogenous VC (as ascorbic acid, the reduced form) levels were determined without any derivatization, using a high-performance liquid chromatography system coupled with a photodiode array according to a previous study (Kondo et al., 2006), with some modifications. Between *Glut12* KO mice and wild-type (WT) mice, VC levels differed negligibly in the plasma (Figure 4A), liver (the principal VC factory in mice), kidney (the major VC eliminating organ), and other main tissues expressing *Glut12* (Figures 4B and S3). However, VC levels in the brain, which includes the CP, of *Glut12* KO mice were approximately half of those of WT mice (Figure 4B). This result, together with our *in vitro* results (Figure 2), is consistent with our working hypothesis that Glut12 is involved in VC supplementation from blood to the brain as VC efflux protein 1 (named Vcep1, encoded by *Glut12*) in the CP. Furthermore, VC concentrations in the CSF of *Glut12* KO mice were considerably lower than in WT mice (Figure 4C). In contrast, VC accumulated significantly in the CP of *Glut12* KO mice compared with that in WT mice (Figure 4D). A similar relationship was observed with the calculated brain-to-, CSF-to-, and CP-to-plasma VC concentration ratios (Figures 4E–4G). The CSF-to-plasma VC concentration ratios in WT mice were >1, whereas the values in *Glut12* KO mice were <0.25 (Figure 4F), suggesting that a directional transport process for VC from blood to the CSF was impaired in *Glut12* KO mice.

In addition, although we have previously found that Glut12 could have a physiological role as a hepatic urate transporter (Toyoda et al., 2020a), current results (Figures 4B and 4E) suggest that Glut12 as a VC transporter might have little effect on apparent (net) VC handling in the liver. Regarding this point, a plausible explanation will be the presence of more influential VC transporter(s) than Glut12 in the liver. Given a previous study (Corpe et al., 2010) showing that hepatic accumulation of 6-bromo-6-deoxy-L-ascorbate (a specific substrate for SVCTs) was drastically decreased in *Svct1* KO mice compared with WT mice, together with the fact that the liver is the organ primarily responsible for VC synthesis in mice, hepatic VC levels may strongly depend on Svct1-mediated VC transport and/or VC production than Glut12-mediated transport. Future investigations are needed into how Glut12 can concert with VC-related proteins including SVCTs in other organs/tissues than the CP.

We observed that the brain of adult *Glut12* KO mice weighed less than that of WT mice, although this difference was not large. This phenotype was not observed in young mice (Figures 4H and S4), implying the importance of continuous VC supply to the brain during the adolescence phase for the maintenance of brain volume. As the brain has the highest VC levels among the major tissues in the body, and as VC participates in epigenetic regulation as an essential cofactor for enzymatic reactions (Young et al., 2015), VC may influence brain development in a period of growth. Together, our observations may partially explain sensorimotor deficits reported in systemic VC-deficient mice models that lack the ability of synthesizing VC (Harrison et al., 2008). In addition, histological analyses showed that at least the hippocampus of adult *Glut12* KO mice was smaller than that of adult WT mice (Figure 4I). However, whether these phenotypes were because of the effect of *Glut12* disruption on cerebral functions remains unclear. Addressing this issue will require conducting a battery of comprehensive behavioral tests using *Glut12* KO mice with a genetic background for VC biosynthesis deficiency (Kondo et al., 2006; Maeda et al., 2000) (similar to that in humans), which will enable the management of internal VC levels by intake amounts. Besides, the *Glut12* KO mouse could be a useful model for brain-specific VC depletion. Thus, our results will pave the way for new strategies that will improve our understanding regarding the pathophysiological impact of brain VC levels on cerebral disorders.

Before closing, a possibility that DHA might also be secreted from CP epithelial cells into the CSF warrants mention. Theoretically, when ascorbate (reduced form of VC) is oxidized within cells, before it is reduced (regeneration process of ascorbate that readily occurs in cells), the generated DHA may be released into extracellular space, which provides another route for VC efflux from cells. However, already-characterized DHA transporters such as Glut1 and Glut3 were not detected in the CP in a previous report (Table S1) and another recent report proposed the localization of Glut1 on the basal side with Svct2, but not the apical side, of CP epithelial cells in the brain of mouse (Ulloa et al., 2019). Moreover, given that ascorbate is the predominant form of cellular VC, at the very least over 80% of VC reportedly exists as the reduced form within cells (Foyer and Noctor, 2011; May et al., 2003; Toutain et al., 1997), deficiency in the Glut12-mediated VC export should be the most plausible explanation of phenotypes observed in *Glut12* KO mice, decrease and increase of VC levels in CSF and the CP, respectively (Figure 4). Thus, this GLUT12-mediated route (Figure 4J) should have great contribution to maintain adequate VC levels in the brain, which is consistent with a previous study demonstrating extremely low (undetectable) VC levels in the brain of *Svct2* KO mice (Sotiriou et al., 2002). On the other hand, as another route crossing the blood-brain barrier, GLUT1-mediated DHA transport has been discussed (Agus et al., 1997; Huang et al., 2001; May, 2012). Although this route remains to be speculative given the lack of investigations using *Glut1*-deficient *in vivo* models, contrary to the route crossing blood-CSF barrier as demonstrated in this study, addressing how physiological conditions influence the differential utilization of these VC delivery routes will be a future issue.

## Conclusion

We successfully identified GLUT12 as a VC efflux protein, named VCEP1, the dysfunction of which significantly decreases the VC levels in the brain, with minimal effects on the plasma VC concentrations. Our findings improve the understanding of VC regulation in the body by unraveling the long-standing mystery in VC handling. Overall, this study is a hypothesis-driven identification of a physiologically important transporter. Our strategy may be applied for identifying other unsung transporters involved in the regulation of substances essential for our body.

### Limitations of the study

Our study has certain limitations. We observed that Glut12 is a physiologically important VC transporter involved in VC accumulation in the brain (Figure 4J). Our data strongly suggest that Glut12 must function, subsequently after Svct2-mediated basal VC uptake, on the apical membrane of CP epithelial cells; however, this polarization should be verified in the future. In addition, despite the dearth of clinical information, we assumed that similar to murine Glut12, human GLUT12 also regulates the VC penetration process in the blood-CSF barrier. In this regard, a microarray dataset (GSE110226) (Stopa et al., 2018) demonstrates the strong expression of *GLUT12* and *SVCT2* in the CP of humans. Moreover, further studies are required to determine the mechanism underlying the *Glut12* KO-mediated decrease in brain weight. Also, *in vivo* experiments in this study were limited to addressing only male. A previous study reported that despite significantly higher plasma VC concentrations in female than male WT mice, there was no significant difference in the VC levels in the brain (Kuo et al., 2004). As such results imply a potential difference in the VC distribution into the brain between male and female, it will be a future issue to examine whether there may be gender differences in the Glut12-mediated VC delivery into the brain and related phenotypes.

## STAR★Methods

### Key resources table

**Table:** 

REAGENT or RESOURCE	SOURCE	IDENTIFIER
**Antibodies**		

Rabbit polyclonal anti-EGFP	Life technologies	Cat# A11122; RRID: AB_221569
Rabbit polyclonal anti-α-tubulin	Abcam	Cat# ab15246; RRID: AB_301787
Donkey anti-rabbit IgG-horseradish peroxidase (HRP)-conjugate	GE Healthcare	Cat# NA934V; RRID: AB_772206

**Chemicals, peptides, and recombinant proteins**		

Ascorbic acid, L-[1-^14^C]-(Vitamin C)	PerkinElmer	Cat# NEC146
L(+)-ascorbic acid	Wako	Cat# 012-04802; CAS: 50-81-7
[8-^14^C]-Uric acid (53 mCi/mmol)	American Radiolabeled Chemicals	Cat# ARC0513
Acyclovir	FUJIFILM Wako Pure Chemical Industries	Cat# 019-17421; CAS: 59277-89-3
Polyethelenimine “MAX” (PEI-MAX)	Polysciences	Cat# 24765; CAS: 49553-93-7

**Critical commercial assays**		

Pierce™ BCA Protein Assay Reagent A & B	Thermo Fisher Scientific	Cat# 23223, 23224
PureLink™ HiPure Plasmid Filter Midiprep Kit	Thermo Fisher Scientific	Cat# K210015
ReverTra ace qPCR RT master mix	TOYOBO	Cat# FSQ-201
SYBR® GreenER™ qPCR SuperMix Universal	Thermo Fisher Scientific	Cat# 11762-500

**Deposited data**		

The Allen mouse brain atlas	Lein et al. (2007)	https://mouse.brain-map.org/
Expression profiling by array: Comparative transcriptomics of choroid plexus in Alzheimer's disease, Huntington's disease and frontotemporal dementia: Implications for CSF homeostasis and dynamics	Stopa et al. (2018)	GEO: GSE110226

**Experimental models: Cell lines**		

Human: HEK293 cells	Toyoda et al. (2016)	N/A
Human: HepG2 cells	Yamanashi et al. (2012)	N/A
Mouse: Hepa1-6 cells	Yamamoto et al. (2017)	N/A
Dog: MDCKII cells	Toyoda et al. (2016)	N/A

**Experimental models: Organisms/strains**		

Mouse: Glut12^8del^	This paper	N/A
Mouse: Glut12^128del^	This paper	N/A
Mouse: B6; 129S7-Uox^tm1Bay^/J	The Jackson Laboratory: Toyoda et al. (2020a)	JAX: 0022238
Mouse: C57BL/6J	Japan SLC	C57BL/6JJmsSlc

**Oligonucleotides**		

A full list of primers	This paper	See Tables S2 and S3
sgRNA for knockout of *Glut12* gene	Toyoda et al. (2020a)	See STAR methods

**Recombinant DNA**		

The complete human GLUT12 cDNA	Toyoda et al. (2020a)	NCBI: NM_145176
The complete mouse Glut12 cDNA	Toyoda et al. (2020a)	NCBI: NM_178934
The complete human SVCT2 cDNA	This paper	NCBI: NM_005116
The complete mouse Svct2 cDNA	This paper	NCBI: NM_018824

**Software and algorithms**		

Excel 2016	Microsoft	https://products.office.com/ja-jp/home
Statcel4 add-in software	OMS Publishing	http://www.oms-publ.co.jp/
GraphPad prism 8	GraphPad Software	https://www.graphpad.com/scientific-software/prism/
MassLynx NT software version 4.1	Waters	https://www.waters.com/waters/ja_JP/MassLynx-Mass-Spectrometry-Software/
ImageJ	NIH	https://imagej.nih.gov/ij/download.html

### Resource availability

#### Lead contact

Further information and requests for resources and reagents should be directed to, and will be fulfilled by, the Lead Contact, Tappei Takada (tappei-tky@umin.ac.jp).

#### Materials availability

Certain materials may be subject to Material Transfer Agreements from The University of Tokyo Hospital, or the original providing entity.

### Experimental model and subject details

#### Animals

All animal experiments were performed according to methods approved by the Institutional Animal Care and Use Committee of The University of Tokyo. All animals received humane care according to the criteria outlined in the Guide for the Care and Use of Laboratory Animals prepared by the National Academy of Sciences and published by the National Institutes of Health.

*Glut12* knockout (KO) mice (Glut^8del^ and Glut^128del^ alleles as *Glut12* KO #1 and *Glut12* KO #2, respectively) were obtained by crossing *Glut12/urate oxidase* (*Uox*) hetero double knockout (DKO) mice on a C57BL/6J genetic background generated using the CRISPR-Cas9 system (Nakao et al., 2016) in our previous study (Toyoda et al., 2020a), with wild-type (WT) C57BL/6J mice (Japan SLC, Shizuoka, Japan). In brief, single-guide (sg) RNAs for the *Glut12* disruption were designed using CRISPRdirect (https://crispr.dbcls.jp/, accessed December 2015); each sgRNA was evaluated using an EGxxFP system (Mashiko et al., 2014)—HEK293 cells transiently co-transfected with sgRNA/pX330, genomic fragments containing the sgRNA target sequence (∼430 bp)/pCAG-EGxxFP, and pCAG-mRFP, simultaneously. Based on the *in vitro* analyses, we selected the target sequence used for genome editing: 5′-cctcatcggggcattcctcgcct-3′. In brief, a murine genomic fragment containing sgRNA target sequence was inserted between EGFP fragments of the pCAG-EGxxFP plasmid. The resulting plasmid was co-transfected with sgRNA/pX330 plasmid (for double expression of sgRNA and Cas9) and pCAG-RFP plasmid (a positive control for plasmid transfection) into HEK293 cells. Forty-eight hours after transfection, the cells were subjected to confocal laser scanning microscopy. In this system, when the target sequence in the pCAG-EGxxFP plasmid is digested by sgRNA-guided Cas9, reconstituted EGFP protein is expressed in the cells, as shown in the main body.

Next, the synthetic sgRNA (FASMAC, Kanagawa, Japan) and *in vitro* transcribed Cas9 mRNA were microinjected into mouse zygotes fertilized *in vitro* (WT eggs × *Uox* KO sperm). Surviving microinjected embryos were transferred into the oviducts of 0.5-day-post-coitum recipient mice. We obtained eight male mice with frameshift mutations in *Glut12*. To confirm germ-line transmission and isolate the single mutated *Glut12* allele, the founder mice were mated with WT mice. Restoration of the *Uox* KO allele in *Glut12* KO mice was confirmed with PCR-based genotyping for *Uox* KO mice [originally obtained from the Jackson Laboratory (Bar Harbor, ME, USA; Stock no.: 002223, B6; 129S7-Uox^tm1Bay^/J) (Wu et al., 1994)], using genomic DNA isolated from ear punch biopsy with hot NaOH, conducted according to the Jackson Laboratory's instructions. Finally, to generate two *Glut12* KO lines by crossing of the mice, we focused on the isolated Glut12^8del^ and Glut12^128del^ alleles derived from different founder mice; these two alleles had 8 and 128 base deletions, respectively, in exon 2 of *Glut12*, and both mutations caused a frame-shift. In this study, we designated mice with homozygous Glut^8del^ and Glut^128del^ alleles as *Glut12* KO #1 and *Glut12* KO #2, respectively. Both KO lines were fertile and capable of giving birth. In addition, we had confirmed in our previous study using *Glut12/Uox* DKO mice (Glut^8del^; Uox^tm1Bay^ and Glut^128del^; Uox^tm1Bay^ alleles as *Glut12/Uox* DKO #1 and *Glut12*/*Uox* DKO #2, respectively), that each frameshift mutation disrupted Glut12 function (Toyoda et al., 2020a).

During the generation of the *Glut12* KO mice, to identify the mutant alleles, genomic fragments containing the *Glut12* target site were amplified by PCR from murine genomic DNA extracted from ear punch biopsy. The PCR products were either cloned with pGEM-T Easy vector and sequenced, or treated with ExoSAP-IT (Affymetrix, Santa Clara, CA, USA) and subjected to direct sequencing. *Glut12* genotypes in the *Glut12* KO mice of F4 or later generations, which were subjected to biochemical measurements, were determined using specific PCR primer sets. PCRs were conducted using GoTaq green PCR master mix (Promega). Amplicons were separated by agarose gel electrophoresis. For genotyping, genomic DNA from each mouse was used as a template; all primers used (1 μM in each reaction mixture) are listed in Table S2. The mice used in this study were males of 6–16 weeks of age, which were maintained on a standard FR-1 diet (Funabashi Farm, Chiba, Japan) with *ad libitum* water, under a 12-h light/dark cycles.

Specimen collection was conducted as follows. Spot urine samples were collected on a plastic wrap sheet and transferred to new tubes. Mice were then anesthetized by intra-peritoneal injection of urethane (1.25 g/kg body weight). Using a glass capillary tube (Calibrated Micropipette 50 μL; Drummond Scientific Company, Broomall, PA, USA), CSF was obtained from the murine brain according to a previous report (Liu and Duff, 2008). Blood was taken from the jugular veins using heparinized syringes and centrifuged at 15,000 × *g* at 4°C for 3 min. The supernatant (plasma) was collected. Immediately after euthanasia, tissues were excised, weighed, and rapidly frozen in liquid nitrogen. In the case of choroid plexus isolation, the lateral ventricular choroid plexus was rapidly and carefully dissected out from the extracted brain under microscopy, and the remaining brain tissue (as others) was also subjected to the following procedures. All specimens were stored at −80°C until further processing.

### Cell culture

HEK293 cells were maintained in Dulbecco's Modified Eagle's Medium (Nacalai Tesque, Kyoto, Japan) supplemented with 10% fetal bovine serum (Biowest, Nuaillé, France), and 1% penicillin-streptomycin (Nacalai Tesque), 2 mM L-Glutamine (Nacalai Tesque), and MEM Non-Essential Amino Acid (Life Technologies, Tokyo, Japan) at 37°C in a humidified atmosphere of 5% (v/v) CO_2_ in air. Madin-Darby canine kidney II (MDCKII) cells were maintained in Dulbecco's Modified Eagle's Medium supplemented with 10% fetal bovine serum, and 1% penicillin-streptomycin, 2 mM L-Glutamine at 37°C in a humidified atmosphere of 5% (v/v) CO_2_ in air.

Vector plasmids encoding each transporter, or mock were transfected into HEK293 cells using polyethyleneimine “MAX” (PEI-MAX) (Polysciences, Warrington, PA, USA) (Miyata et al., 2016). In brief, HEK293 cells were seeded onto collagen-coated glass-bottom dishes (Matsunami Glass, Tokyo, Japan) for confocal microscopic observations or 12-well cell-culture plates for transport assays at a concentration of 0.92 × 10^5^ cells/cm^2^. Twenty-four hours after seeding, cells were transiently transfected with the respective plasmid vectors using PEI-MAX (0.75 μg plasmid/3.75 μL of PEI-MAX/well). The medium was replaced with fresh medium after the first 24 h of incubation.

With z-stack microscopic observation for polarized cells, MDCKII cells were seeded onto collagen-coated glass-bottom dishes at a concentration of 1.31 × 10^5^ cells/cm^2^, and then transiently transfected with the respective plasmid vectors using PEI-MAX (2.0 μg plasmid/10 μL of PEI-MAX/dish). The cells were further incubated for 48 h. The culture medium was replaced with a fresh one after the first 24 h of incubation.

### Methods details

#### Materials

Critical materials and resources used in this study are summarized in Key resources table. All other chemicals used were commercially available and were of analytical grade.

#### Plasmid constructions

The full-length of the wild-type (WT) human GLUT12/SLC2A12 (NCBI accession no. NM_145176) open reading frame (ORF) and the full-length of the WT mouse Glut12/Slc2a12 (NCBI accession no. NM_178934) ORF were cloned and inserted into a pEGFP-N1 vector (Clontech Laboratories, Palo Alto, CA, USA) for EGFP-tagged GLUT12 or Glut12 expression in our previous study (Toyoda et al., 2020a). Using a site-directed mutagenesis technique, two expression vectors for frameshift mutants (8-bp deletion and 128-bp deletion) of Glut12 found in each *Glut12* KO line were constructed with the pEGFP-N1 vector, according to our previous study (Toyoda et al., 2016).

The full-length of the WT human SVCT2/SLC23A2 (NCBI accession no. NM_005116) ORF and mouse Svct2/Slc23a2 (NCBI accession no. NM_018824) ORF were PCR-amplified from a total cDNA library of human hepatoma HepG2 cells and mouse hepatoma Hepa1-6 cells, respectively. After cloning into pGEM-T Easy vector (Promega, Fitchburg, WI, USA), each ORF was inserted into the pEGFP-N1 vector plasmid. For removal of the original termination codon and in-frame fusion with a downstream EGFP protein (i.e., SVCT2-EGFP expression), the site-directed mutagenesis technique was successfully employed. Functional confirmation of these expression vectors is described later.

All plasmid constructs were confirmed by full sequencing using BigDye® Terminator v3.1 (Applied Biosystems, Foster City, CA, USA) on an Applied Biosystems® 3130 Genetic Analyzer (Applied Biosystems), according to the manufacturer’s protocol. All plasmids used in the experiments were from the same lot.

#### Whole cell lysate preparation and immunoblotting

Forty-eight hours after the plasmid transfection, HEK293 cells were washed with ice-cold phosphate-buffered saline without potassium [PBS (−)] twice, and were lysed with an ice-cold RIPA lysis buffer [50 mM Tris-HCl, pH 7.4, 150 mM NaCl, 0.1% sodium dodecyl sulfate (SDS), 0.5% sodium deoxycholate, 1% NP-40, 1 mM phenylmethylsulfonyl fluoride, and a Protease Inhibitor Cocktail for General Use (Nacalai Tesque)]. The solution was centrifuged at 15,000 × *g* at 4°C for 10 min and the resulting supernatant (whole cell lysate) was collected in a new tube. The protein concentration of the whole cell lysate was quantified using a Pierce™ BCA Protein Assay Kit (Thermo Fisher Scientific, Carlsbad, CA, USA) with BSA as a standard, according to the manufacturer's protocol.

Immunoblot analyses were performed as described in our previous report (Toyoda et al., 2020a), with minor modifications. Briefly, whole cell lysate samples were separated by SDS polyacrylamide gel electrophoresis and transferred to an Immobilon-P PVDF membrane (Millipore, Bedford, MA, USA) by electroblotting at 15 V for 60 min. For blocking, the membrane was incubated in Tris-buffered saline containing 0.05% Tween 20 and 3% BSA (TBST-3% BSA). Blots were probed with appropriate antibodies (Key Resources Table), and then an HRP-dependent luminescence was developed with ECL™ Prime Western Blotting Detection Reagent (GE Healthcare, UK). Immunocomplexes were detected using a multi-imaging Analyzer Fusion Solo 4™ system (Vilber Lourmat, Eberhardzell, Germany).

#### Confocal microscopy

For confocal laser-scanning microscopy, specimens were prepared according to our previous study (Toyoda et al., 2020b), with minor modifications. Briefly, 48 h after transfection, cells were fixed with ice-cold methanol and then treated with 0.2 mg/mL RNase in PBS (−) to degrade intracellular RNAs. After washing with PBS (−), the cells were treated with TO-PRO-3 Iodide (Molecular Probes, Eugene, OR, USA) staining for 20 min at room temperature in the dark. After visualizing the nuclei, the cells were washed with PBS (−) five times and mounted on Fluorescence Mounting Medium (Agilent, Santa Clara, CA, USA). To analyze the localization of EGFP-fused transporter proteins, fluorescence was observed using the FV10i Confocal Laser Scanning Microscope (Olympus, Tokyo, Japan) or the FV1000 Confocal Laser Scanning Microscope (Olympus).

#### RNA extraction and qPCR

Total RNA was extracted from murine tissues or culture cells using the RNAiso Plus Reagent (TaKaRa Bio, Shiga, Japan), according to the manufacturer's protocol. Reverse transcriptional reaction and subsequent qPCR were performed using ReverTra Ace qPCR RT Master Mix (TOYOBO, Osaka, Japan) and SYBR GreenER™ qPCR SuperMix Universal (Thermo Fisher Scientific), respectively; the qPCR signals were monitored with an Eco real-time PCR system (Illumina, San Diego, CA, USA) and the associated software (Toyoda et al., 2019). The expression levels of each gene were normalized to those of β-actin. The sequences of the primers used are shown in Table S3.

#### Vitamin C transport assay using HEK293 cells

To determine the VC transport activities of human GLUT12 and mouse Glut12, cell-based VC uptake assays using human GLUT12-and mouse Glut12-expressing HEK293 cells were conducted as described in our previous studies (Miyata et al., 2016; Toyoda et al., 2020a), with some modifications. In a series of uptake assays, SVCT2 or Svct2 was employed as a positive control for VC transport. In brief, 48 h after plasmid transfection, the cells were washed twice with an indicated transport (TP) buffer (the composition of each buffer is summarized in Table S4) and pre-incubated in TP buffer at 37°C for 15 min. Then, the TP buffer was replaced with pre-warmed fresh TP buffer containing [1-^14^C]-VC (L-ascorbic acid) (7.3 mCi/mmol; PerkinElmer, Waltham, MA, USA) at indicated concentrations, and the cells were further incubated for the indicated periods. Unless otherwise noted, the [1-^14^C]-VC concentration and incubation period in the present study were 20 μM and 10 min, respectively. Subsequently, the cells were washed with ice-cold TP buffer twice and then lysed with 500 μL of 0.2 M NaOH on ice under gentle shaking for 1 h. The lysates were neutralized with 100 μL of 1 M HCl. Then, the radioactivity in the lysate was measured using a liquid scintillator (Tri-Carb 3110TR; PerkinElmer). The protein concentrations in the lysates were determined using the Pierce™ BCA Protein Assay Kit, as described above.

The VC transport activity was calculated as incorporated clearance (μL/mg protein/min) = (incorporated level of VC [DPM/mg protein/min]/VC level in the incubation mixture [DPM/μL]). Human GLUT12- and mouse Glut12-mediated VC transport activities were calculated by subtracting the VC transport activity of mock cells from that of human GLUT12- and mouse Glut12-expressing cells, respectively.

#### Urate transport assay using HEK293 cells

To confirm the transporter function of human GLUT12 and mouse Glut12, cell-based urate uptake assays using human GLUT12- and mouse Glut12-expressing HEK293 cells were conducted as described in our previous study (Toyoda et al., 2020a). In brief, cells were incubated in Krebs–Ringer buffer (pH 5.4) containing 10 μM [8-^14^C]-urate (American Radiolabeled Chemicals, St. Louis, MO, USA) for 10 min, then the urate transport activity was calculated as incorporated clearance, as described above.

#### Vitamin C efflux assay using HEK293 cells

To determine the VC efflux activities of human GLUT12 and mouse Glut12, cell-based VC efflux assays using human GLUT12- and mouse Glut12-expressing HEK293 cells with co-expression of human SVCT2 and mouse Svct2 were conducted using 24-well cell-culture plates, respectively. In a series of efflux assays, to equalize the amount of plasmid used for transient transfection among the wells, a mock vector was used. Notably, human SVCT2 or mouse Svct2 was employed to achieve sufficient VC incorporation into the cells. Since they are sodium-dependent vitamin C transporters, cells were incubated with sodium-free transport buffer at the efflux phase to evaluate the cellular function of human GLUT12 and mouse Glut12 in the absence of that of human SVCT2 and mouse Svct2, respectively. The details are as follows.

First, for the uptake phase, 48 h after double plasmid transfection, the cells were washed twice with Krebs–Ringer buffer containing 5 μM non-radiolabeled VC and pre-incubated in the Krebs–Ringer buffer at 37°C for 15 min. Next, the Krebs–Ringer buffer was replaced with pre-warmed fresh Krebs–Ringer buffer containing 20 μM [1-^14^C]-VC, and the cells were further incubated at 37°C for 40 min to incorporate radio-labeled VC into the cells.

Next, for the efflux phase, the cells were washed twice on ice with ice-cold sodium-free transport buffer [Na^+^-free TP buffer] containing 5 μM non-radiolabeled VC to remove the remaining extracellular radio-labeled VC. The buffer was then replaced with 300 μL of the pre-incubated Na^+^-free TP buffer (time: 0 min), and the cells were further incubated with at 37°C for 105 min. At 15 min and 45 min, 100 μL of the incubation buffer was collected and the same volume of fresh buffer was added to maintain the total volume of incubation buffer in the well.

Finally, the cells were washed twice with ice-cold Na^+^-free TP buffer and then lysed with 250 μL of 0.2 M NaOH on ice under gentle shaking for 1 h. The lysates were neutralized with 50 μL of 1 M HCl. Then, the radioactivity in the lysate and collected buffer (at 15, 40, and 105 min) was measured using a liquid scintillator (Tri-Carb 3110TR). The protein concentrations in the lysates were determined using the Pierce™ BCA Protein Assay Kit as described above. The VC efflux values were evaluated as efflux ratio (%) = (media-released radio-labeled VC in the well [DPM/mg protein]/incorporated radio-labeled VC at 0 min [DPM/mg protein] × 100). To calculate the incorporated radio-labeled VC at 0 min, the total amount of media-released radio-activity and intracellular radio-activity at 105 min were summed. The apparent VC efflux activities were calculated as [1-^14^C]-VC secretion rate determined by linear regression analysis (0–45 min) [min/DPM]/cellular [1-^14^C]-VC level at 0 min [DPM/μg protein].

#### Specimen collection and sample preparation

After thawing on ice, each specimen was preprocessed for liquid chromatography-photodiode array (LC-PDA) analysis as follows.

Each plasma or 10 times-diluted urine sample was deproteinized with equal volumes of 10% (w/v) metaphosphate solution containing 1 mM EDTA, and centrifuged at 20,000 × *g* at 4°C for 10 min. The resulting supernatant was diluted with equal volumes of 25 mM phosphate buffer (pH 2.1) containing 60 μM acyclovir (Wako Pure Chemical Industries, Osaka, Japan) as an internal control. All volumes of collected CSF (a several μL/mouse) were mixed with 7.5 μL of 120 μM acyclovir solution, then up to 15 μL 1 mM EDTA solution. The mixture was deproteinized with 15 μL of 10% (w/v) metaphosphate solution containing 1 mM EDTA, and centrifuged at 20,000 × *g* at 4°C for 10 min. The resulting supernatant was used for subsequent LC-PDA analysis.

For all tissues except for the choroid plexus, each extracted tissue was homogenized with 14 volumes of ice-cold 5.4% (w/v) metaphosphate solution containing 1 mM EDTA using an ice-cold Physcotron homogenizer (Microtec, Chiba, Japan), and centrifuged at 20,000 × *g* at 4°C for 10 min to remove debris and cell nuclei, as well as for deproteinization. The resulting supernatant was diluted with equal volumes of 25 mM phosphate buffer (pH 2.1) containing 60 μM acyclovir to obtain analytical samples. The choroid plexus was homogenized in 20 μL of ice-cold PBS (−) using a BioMasher II (Nippi, Tokyo, Japan) and centrifuged at 20,000 × *g* at 4°C for 10 min. For deproteinization, 10 μL of the resulting supernatant was mixed with 15 μL of 10% (w/v) metaphosphate solution containing 1 mM EDTA and 5 μL of 180 μM acyclovir, and then centrifuged at 20,000 × *g* at 4°C for 10 min. The resulting supernatant was used for subsequent LC-PDA analysis.

#### Experimental measurement of vitamin C levels

Vitamin C levels in plasma, urine, CSF, and tissues were measured by LC-PDA analysis according to a previous report (Kondo et al., 2006), with some modifications.

In brief, all samples were analyzed on an ACQUITY UPLC PDA Detector (Waters, Milford, MA, USA) coupled with an ACQUITY UPLC System (Waters). A volume of 5 μL of each sample was injected into a CAPCELL PAK ADME S3 column (3 μm, 2.1 × 100 mm; Osaka Soda, Osaka, Japan) and separated. Elution was conducted using a gradient mobile phase (0–4 min: 2% B; 4–5 min: 2–98% B; 5–9 min: 98% B; 9–10 min: 98–2% B; 10–12 min: 2% B) of 25 mM phosphate buffer (pH 2.1) (A) and methanol (B) at a flow rate of 300 μL/min. The column was maintained at 50°C. During the separation, the PDA spectrum was obtained, and 243 nm was chosen for measurement of VC and acyclovir. Calibration curves for the analyte were generated from a series of murine plasma spiked with standard solutions of VC (L-ascorbic acid). Peak analyses were conducted using MassLynx NT software v4.1 (Waters).

#### Brain histology

Brain histology was performed according to previous studies (Kasahara et al., 2019; Senturk et al., 2011). In brief, the mice were perfused with cold PBS (−) followed by 4% paraformaldehyde (PFA) phosphate buffer solution (FUJIFILM Wako Pure Chemical Industries, Osaka, Japan). Brain samples were postfixed with 4% PFA overnight and rinsed three times with 0.1 M PBS (−). The brain samples were sectioned into 100 μm-thick coronal slices using a DTK-1500 vibratome (Dosaka, Kyoto, Japan), and then incubated with Hoechst in PBS (−) at room temperature for 10 min with agitation to reveal neuronal cytoarchitecture. The sectioned samples were rinsed three times with 0.1 M PBS (−) and embedded in Permafluor (Thermo Fisher Scientific). Images (5–10 brain sections from each animal) were acquired using a BZ-X700 microscope (Keyence, Osaka, Japan), and hippocampal areas were calculated using the ImageJ program (NIH, Bethesda, MD, USA).

### Quantification and statistical analyses

Unless otherwise noted, data are presented as mean ± SEM. All statistical analyses were performed in Excel 2016 (Microsoft, Redmond, WA, USA) with Statcel4 add-in software (OMS Publishing, Saitama, Japan) or GraphPad Prism 8 (GraphPad Software, San Diego, CA). Different statistical tests were used for different experiments as described in the figure legends. Briefly, when analyzing multiple groups, the similarity of variance between groups was compared using Bartlett's test. If the test for homogeneity of variance was passed, a parametric Tukey–Kramer multiple-comparison test was used; otherwise, a non-parametric Steel–Dwass test was used. In the case of a single pair of quantitative data, after comparing the variances of a set of data with an *F*-test, unpaired Student's or Welch's *t* test were performed. Statistical significance was defined in terms of *P* values less than 0.05 or 0.01.

Each experiment was designed to use the minimum number of mice or samples required to obtain informative results and sufficient material for subsequent studies. No specific statistical test was used to pre-determine the sample sizes that were empirically determined in the current study. All experiments were monitored in a non-blinded fashion. Samples that had undergone technical failure during processing were excluded from analyses. The numbers of biological replicates (*n*) are described in the figure legends.

## Data Availability

Data: Data supporting the findings of this study are included in this published article and its Supplemental information or are available from the corresponding authors on reasonable request. Code: This paper does not report original code. Other items: Any additional information required to reanalyze the data reported in this paper is available from the lead contact upon request.
