# Supplementary material for: Identification of an exporter that regulates vitamin C supply from blood to the brain
Source: iScience. 2022 Jan 13;25(1):103642. doi: 10.1016/j.isci.2021.103642 (PMC8786643; doi:10.1016/j.isci.2021.103642)
Supplement: Document S1. Figures S1–S4 and Tables S1–S4 [file mmc1.pdf]

iScience, Volume *25*

Supplemental information

Identification of an exporter that regulates vitamin C supply from blood to the brain

Hiroshi Miyata, Yu Toyoda, Tappei Takada, Toshimitsu Hiragi, Yu Kubota, Ryuichiro Shigesawa, Ryuta Koyama, Yuji Ikegaya, and Hiroshi Suzuki

SUPPLEMENTAL FIGURES

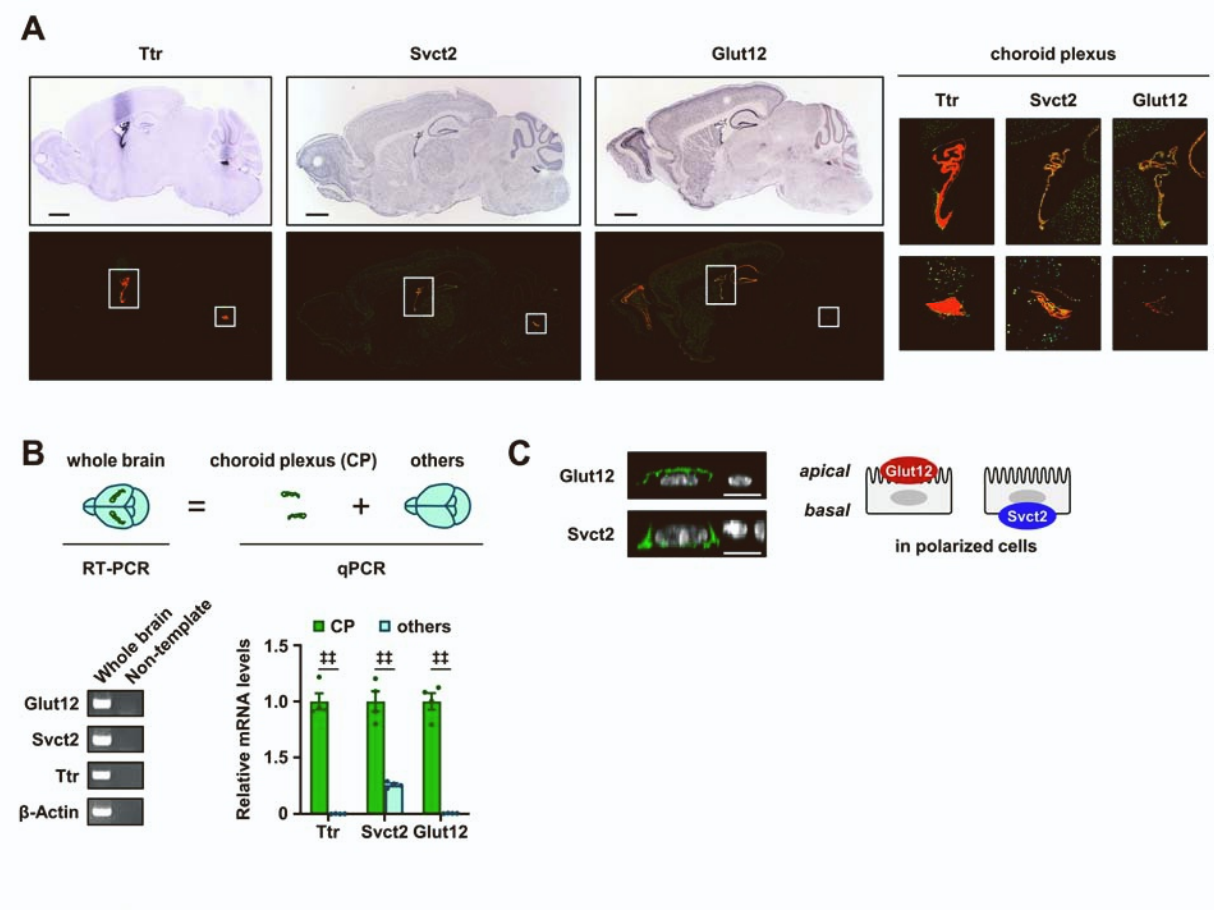

Figure S1 │Glut12 is expressed in the choroid plexus in murine brain. Related to Figure 1.
(A) Representative images of *in situ* hybridization (IHC, *upper*; Expression, *lower*) for Glut12, Svct2, and transthyretin (Ttr) obtained from open source data generated by the Allen Mouse Brain Atlas (https://mouse.brain-map.org/) (E. S. Lein *et al.*, *Nature* 445, 168-176, 2007). Ttr is a choroid plexus (CP) marker. Magnified images of the CP in the inferior horn of the lateral ventricle and the fourth ventricle outlined by white rectangles and squares, respectively, are also shown. Bars, 1,199 µm. **(B)** Glut12 expression in the CP detected by reverse transcription (RT)-PCR. RT-PCR analyses for the whole brain of the wild-type mouse confirmed that Glut12 and Svct2 were expressed in the brain similarly to Ttr. Prior to quantitative PCR (qPCR) analyses, the CP in the lateral ventricle of the brain of wild-type mice was carefully extracted from the brain. Then, the expression levels of each gene were quantitatively examined in the CP and the remaining parts of the brain (Others). In qPCR analyses, β-actin mRNA was used as an internal control; fold-changes in the expression levels of each gene were normalized to those in the CP. Like Ttr, Glut12 was expressed more strongly in the CP than in other parts of the brain. Data are mean ± SEM, $n = 4$. ‡‡ $P < 0.01$ (paired *t*-test). **(C)** Apical localization of Glut12 in polarized MDCKII cells 48 h after transfection. Contrary to the Svct2 tagged with EGFP (Svct2-EGFP) showing basolateral localization, Glut12 (Glut12-EGFP) was localized on the apical membrane of the cells. Nuclei were stained with TO-PRO®-3 iodide (gray). Bars, 10 µm. Both panels show the Z-sectioning images.

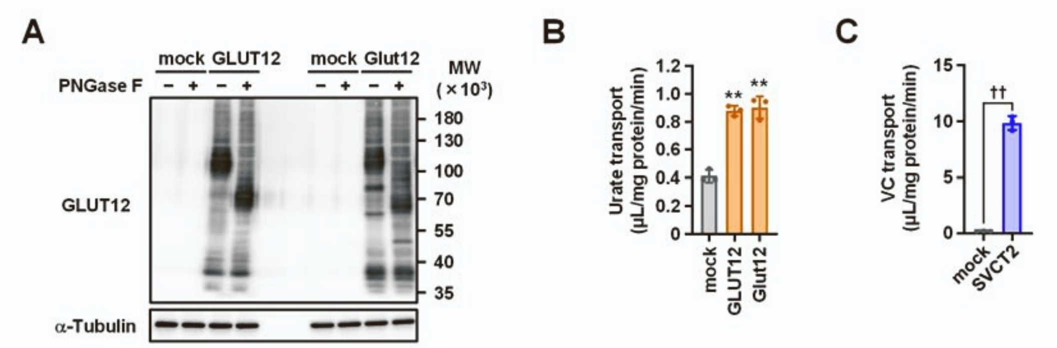

Figure S2 │Confirmation of the functional expression of GLUT12 and Glut12 in HEK293 cells as well as vitamin C transport activities mediated by human SVCT2. Related to Figures 1 and 2.

(A, B) Forty-eight hours after plasmid transfection, cells transiently expressing EGFP-tagged human GLUT12 or mouse Glut12 were subjected to immunoblotting and urate uptake assay. **(A)** Immunoblot detection of GLUT12 and Glut12 proteins in whole cell lysate using an EGFP-antibody. α-Tubulin was used as a loading control. **(B)** Urate transport activities of GLUT12 and Glut12. In this transport assay, cells were incubated in Krebs–Ringer buffer (pH 5.4) containing 10 μM [8-^{14}C]-urate for 10 min. Data are mean ± SD, n = 3. Statistical analyses for significant differences in each group were performed using Bartlett's test, followed by Dunnett's test ($^{**}P < 0.01$ *vs.* Mock). **(C)** Forty-eight hours after plasmid transfection, HEK293 cells transiently expressing EGFP-tagged human SVCT2 were subjected to a vitamin C (VC) uptake assay. In this transport assay, cells were incubated in Krebs–Ringer buffer (pH 7.4) containing 20 μM [1-^{14}C]-VC for 5 min. Data are mean ± SD, n = 3. †† $P < 0.01$ (two-sided t-test). Given the substrate specificity of SVCT2 that transports VC, this result indicated that [1-^{14}C]-VC, as it is, was incorporated into SVCT2-expressing cells during the uptake stage in our efflux assay system (**Figure 2A**). In other words, considering the extremely lower background in the mock control cells, accumulation of radioactivity was due mostly to VC.

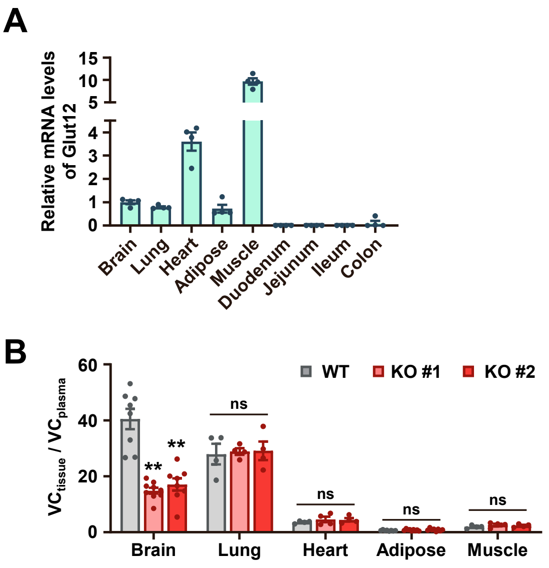

Figure S3 │*Glut12* knockout affected the apparent VC distribution from blood to the brain but not to other main tissues. Related to Figure 4.

(A) Glut12 expression in several tissues of wild-type (WT) mice. β-Actin mRNA was used as an internal control; relative expression levels of Glut12 were normalized to those in the brain. Data are mean ± SEM, $n = 4$. **(B)** Calculated tissue-to-plasma VC concentration ratios. Plasma VC concentrations are shown in **Figure 4A**. Data are mean ± SEM, $n = 8$ (WT), 9 (KO #1), 8 (KO #2) for the brain and adipose tissue, and 4 for the other groups. Statistical analyses for significant differences in each group were performed using Bartlett's test, followed by Dunnett's test ($^{**}P < 0.01$ *vs.* WT. ns, not significantly different).

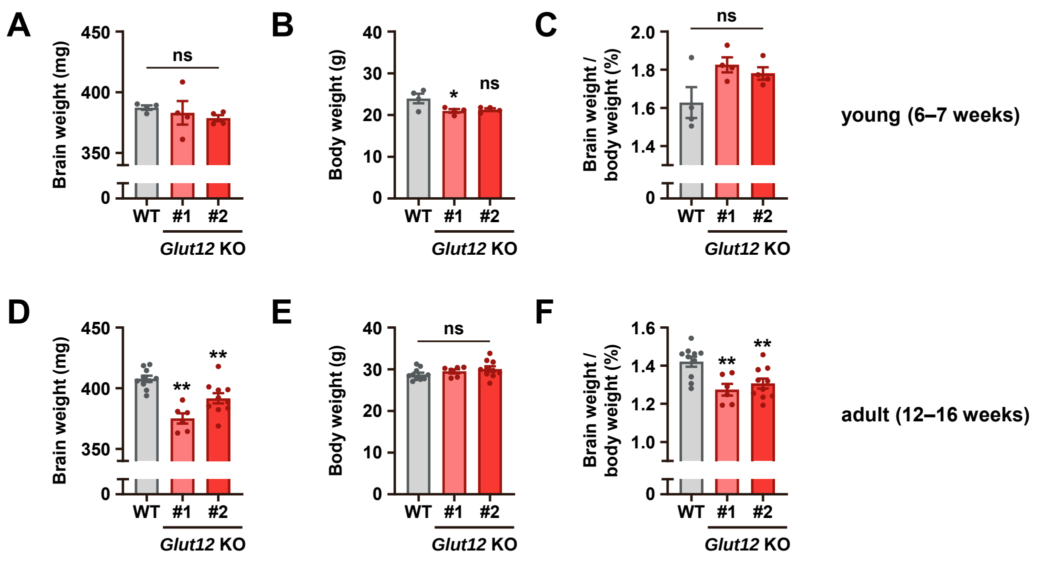

Figure S4 │*Glut12* knockout-mediated decrease of brain weight in adult mice. Related to Figure 4.
The effect of *Glut12* knockout on the brain weight (A, D), body weight (B, E), and brain weight-to-body weight ratio (C, F) in young mice **(A–C)** and adult mice **(D–F)**. The panel of brain weight of adult mice (D) is also shown in the main body in **Figure 4H**. Data are mean ± SEM, $n = 4$ (A–C), and 10 (WT), 6 (KO #1), 10 (KO #2) (D–F). Statistical analyses for significant differences in each group were performed using Bartlett's test, followed by non-parametric Steel test (A) or Dunnett's test (B–F) ($^{*}P < 0.05$; $^{**}P < 0.01$ *vs.* WT. ns, not significantly different).

SUPPLEMENTAL TABLES

Table S1. ***Glut*** **genes highly expressed in the choroid plexus in mice.** Related to Figure 1.

Symbol	Gene	Experiment ID in Allen Brain Atlas	Probe Name	Expression in the Choroid Plexus
Slc2a1	*Glut1*	69873875	RP_050512_02_B12	ND
Slc2a2	*Glut2*	69015679	RP_050315_05_B10	ND
Slc2a3	*Glut3*	1841	RP_040507_02_G12	ND
Slc2a4	*Glut4*	2600	RP_040428_01_C04	ND
Slc2a5	*Glut5*	2602	RP_040526_02_E10	ND
Slc2a6	*Glut6*	69873881	RP_050512_02_C01	ND
Slc2a7	*Glut7*	NA	–	–
Slc2a8	*Glut8*	69873887	RP_050512_02_C02	ND
Slc2a9	*Glut9*	78534486	RP_070620_01_A02	ND
Slc2a10	*Glut10*	100145322	RP_110519_02_C09	ND
Slc2a11	*Glut11*	NA	–	–
Slc2a12	*Glut12*	70302135	RP_050607_03_F08	Highly expressed
Slc2a13	*HMIT*	70228549	RP_050524_03_H06	ND
Slc2a14	*Glut14*	NA	–	–
Slc23a2	*Svct2*	68797840	RP_050215_01_C04	Highly expressed
Ttr	*Ttr*	68632172	RP_050125_01_G11	Highly expressed

NA, not available; ND, not detected.
To explore *Glut* genes expressed in the CP of the murine brain, *in situ* hybridization image data obtained from the Allen Mouse Brain Atlas (https://mouse.brain-map.org/) (E. S. Lein *et al.*, *Nature* 445, 168-176, 2007) were addressed. Representative *in situ* hybridization images for Glut12, Svct2 and Ttr (a choroid plexus marker) are shown in **Figure S1A**.

Table S2. Primer sequences for genotyping of each knockout mouse. Related to Figure 3.

**Primers**	**Nucleotide sequences (5′ to 3′)**	**Amplicon (bp)**
Glut12^{8del}		
Forward (for WT)	gttccctcctcatcggggca	156
Forward (for KO)	gtgagttccctcctcattcc	152
Reverse (common)	ccattataaggagcgtgtag	
Glut12^{128del}		
Forward	ggttggttgtatgtgtgtcc	431 (WT), 303 (KO)
Reverse	ccattataaggagcgtgtag	
Uox		
Forward (for WT)	tcgagacctttgcaatgaacatc	276
Forward (for KO)	cgccttctatcgccttcttgacg	150
Reverse (common)	ttctcatctgctccacctcacag	

WT, wild-type; KO, knockout.

For the genotyping of the *Uox* KO allele, all (three) primers were used in one reaction tube.

Thermal cycling conditions for genotyping were as follows: 95°C for 2 min; 28 cycles of 95°C for 30 s, 58°C for 10 s, 72°C for 15 s; 5 min at 72°C (for KO #1: *Glut12^{8del}*), and 95°C for 2 min; 30 cycles of 95°C for 30 s, 60°C for 30 s, 72°C for 20 s; 5 min at 72°C (for KO #2: *Glut12^{128del}* and *Uox*). Representative band patterns of each amplicon are shown in **Figure 3C**.

Table S3. Primer sequences for qPCR analysis for each gene in *Mus musculus*. Related to STAR Methods.

Gene	Gene name		Sequence 5′ to 3′
β-Actin	Actin, beta	F	ttcaacaccccagccatgtacg
		R	gtggtggtgaagctgtagcc
Glut12	Solute carrier family 2 (facilitated glucose transporter), member 12	F	gctagcaaaggcgaactatgtg
		R	ggtggttacactctgggagc
Svct2	Solute carrier family 23 (nucleobase transporters), member 2	F	gagctgcaggcaggtgatag
		R	gggcttagtcaagccaggag
Ttr	Transthyretin	F	acttggcatttccccgttcc
		R	ccgtggtgctgtaggagtatg

Table S4. Compositions of transport buffers used in the present study. Related to STAR Methods.

	**Final concentrations [mM]**
Krebs–Ringer buffer	
NaCl*	133
KCl	4.93
$MgSO_4$	1.23
$CaCl_2$	0.85
Glucose	5
L-glutamine	5
HEPES	10
MES	10
	pH 5.4, 6.4, 7.4
K-high buffer	
NaCl	None
KCl	145.4
$MgSO_4$	0.8
$CaCl_2$	1.8
Glucose	5
HEPES	25
Tris	25
	pH 7.4
Cl-free buffer	
Na-gluconate	125
K-gluconate	4.8
$MgSO_4$	1.2
Ca-gluconate	1.3
KH_2PO_4	1.2
Glucose	5.6
HEPES	25
	pH 7.4

*NaCl was replaced with choline-Cl for Na^+-free Krebs–Ringer buffer.
